# Association of Implantation Depth With Hemodynamics and Mechanical Consequences in Small Valve-In-Valve TAVR

**DOI:** 10.1016/j.jacasi.2026.07.050

**Published:** 2026-10-06

**Authors:** Bo Fu, Fenlong Xue, Yanhe Ma, Zhengda Chen, Shihong Liu, Hongyue Sun, Zhigang Guo, Qingliang Chen, Nan Jiang

**Affiliations:** a Department of Cardiovascular Surgery, Chest Hospital, Tianjin University, Tianjin, China; b Clinical School of Thoracic, Tianjin Medical University, Tianjin, China; c Department of Radiology, Chest Hospital, Tianjin University, Tianjin, China; d College of Biomedical Engineering, Fudan University, Shanghai, China; e Department of Occupational and Environmental Health, School of Public Health, Tianjin Medical University, Tianjin, China; f Key Laboratory of Prevention and Control of Major Diseases in the Population, Ministry of Education, Tianjin Medical University, Tianjin, China

**Keywords:** hemodynamics, implantation depth, small surgical bioprosthesis, TAVR, valve-in-valve

## Abstract

**Background:**

Valve-in-valve (ViV) transcatheter aortic valve replacement in small surgical bioprostheses is often limited by residual gradients and prosthesis-patient mismatch. Implantation depth is operator-controlled, but depth guidance for different valve combinations remains limited.

**Objectives:**

The authors aimed to evaluate the effects of implantation depth on ViV hemodynamics, leaflet deformation, and pinwheeling in small surgical bioprostheses and to assess the mechanical consequences of THV underexpansion using accelerated wear testing.

**Methods:**

A 21-mm self-expanding transcatheter heart valve was implanted in five 21-mm surgical heart valves at multiple depths in a pulse duplicator. Mean gradient, effective orifice area, regurgitation volume, energy loss, leaflet deformation, and pinwheeling were assessed. Selected constrained ViV configurations underwent 200 million cycles of accelerated wear testing under ISO 5840 conditions.

**Results:**

Implantation depth substantially influenced ViV performance. In 3 porcine surgical valves, the most favorable combined hemodynamic profile occurred within a moderate-to-deep depth window of approximately 13.0 to 14.5 mm, although the optimal value differed across individual hemodynamic and deformation endpoints. Excessive depth increased frame constraint, leaflet distortion, and pinwheeling. Two bovine surgical valves showed mixed or U-shaped responses, consistent with differences in true internal diameter and transcatheter valve oversizing. The selected ViV configurations completed 200 million accelerated wear cycles without gross structural failure under bench conditions.

**Conclusions:**

In small ViV-transcatheter aortic valve replacement, the hemodynamic effects of implantation depth are valve-combination specific. A depth-window strategy may improve acute hydrodynamic performance, but clinical translation requires balancing hemodynamic benefit against leaflet deformation and durability-related uncertainty.

Valve-in-valve (ViV) transcatheter aortic valve replacement (TAVR) has replaced redo surgery as the preferred treatment for failed surgical bioprostheses, largely because of lower perioperative morbidity and better early survival.^1^ As preprocedural planning and fluoroscopic guidance mature, the problems of malposition and coronary obstruction have been partially solved.^2^, ^3^, ^4^ Yet elevated residual gradients and patient-prosthesis mismatch remain unresolved, especially in small bioprosthetic valves.^5^ Bioprosthetic valve fracture helps relieve patient-prosthesis mismatch but introduces extra procedural risk and is incompatible with certain valve types.^6^^,^^7^

Under these constraints, implantation depth—a parameter the operator directly controls—becomes one of the few immediately adjustable procedural variables for optimizing ViV hemodynamics.^8^, ^9^, ^10^ The ideal deployment secures the transcatheter heart valve (THV), positions its uncovered portion above the sewing ring, completely covers the degenerated bioprosthetic leaflets, avoids coronary obstruction, and still delivers optimal hemodynamics.^11^ Self-expanding supra-annular valves tend to produce lower gradients than balloon-expandable ones in small bioprostheses,^5^ but translation of these data across all valve pairings is limited because of substantial design variability. The challenge is particularly pronounced in Chinese patients, in whom the average surgical valve size is only 21 mm. In addition, approximately 70% of implanted surgical bioprostheses are Hancock II valves, which are not amenable to bioprosthetic valve fracture according to national registry data,^12^ and therefore depth remains among the few immediately actionable variables. The VitaFlow valve (MicroPort CardioFlow Medtech Corporation) dominates ViV practice in China and is being adopted in Europe as a representative Chinese transcatheter platform.^13^ Its functional region sits markedly lower than that of the Evolut PRO (Figure 1), yet neither labeling nor the ViV app supplies depth guidance, and rigorous data are absent.^14^

**Figure 1:**
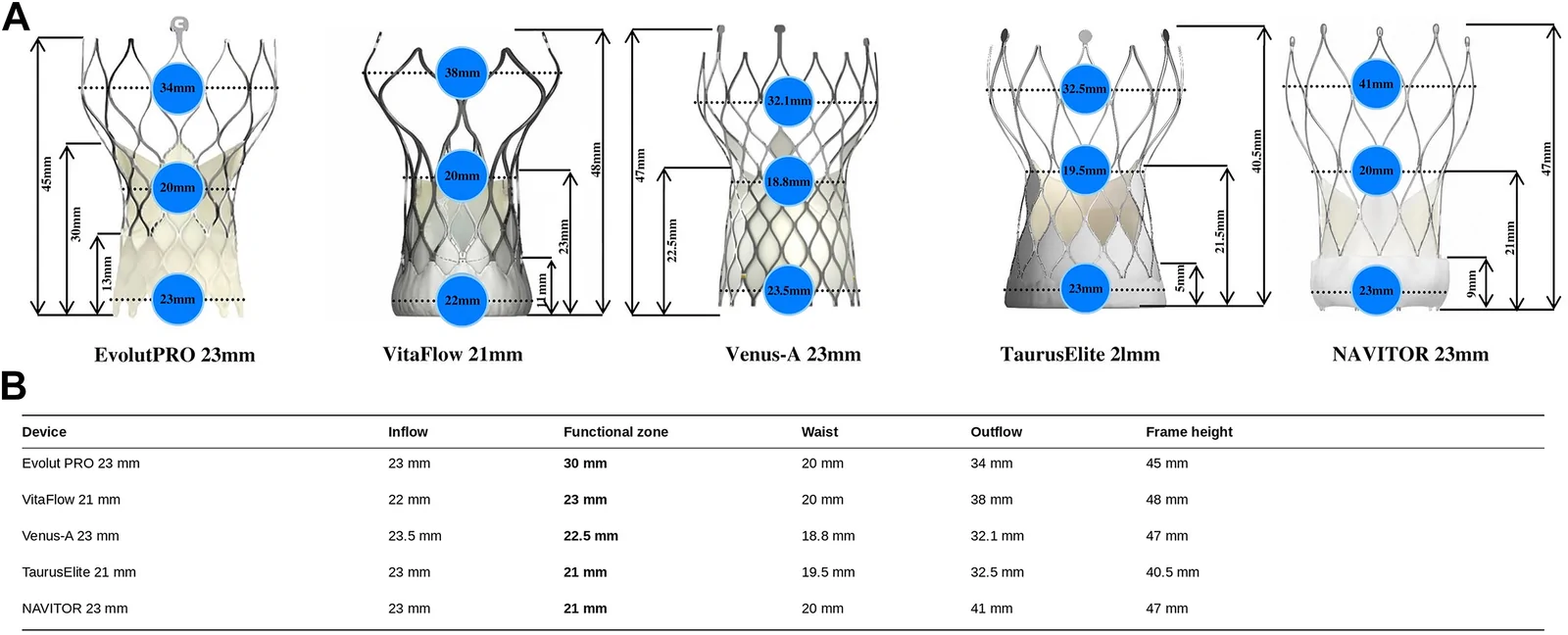
Transcatheter Valve Frame Geometry Structural and dimensional features of 5 transcatheter heart valve platforms are compared. (A) Schematic illustrations show frame configuration and key measurements, including inflow diameter, functional zone diameter, waist diameter, outflow diameter, and frame height. (B) Dimensional differences among valve platforms are summarized to illustrate variation in stent-frame architecture and functional-zone geometry.

This study therefore evaluated the impact of implantation depth on ViV hemodynamics, leaflet deformation, and pinwheeling across 5 21-mm surgical bioprostheses and investigated the mechanical consequences of THV underexpansion using accelerated wear testing.

## Materials and Methods

### THVs and ViV deployment

The 21-mm VitaFlow self-expanding THV was implanted within five types of 21-mm surgical heart valves (SHVs), including Hancock II and Mosaic (Medtronic), Perimount 2800 and Magna Ease (Edwards Lifesciences), and Epic/Biocor (Abbott), whereas the THV commissure posts were aligned with the bioprostheses’ commissure angle. Stepwise changes in implantation depth of the THV were attempted within the SHVs. Implantation depth was determined using computer technology (CT) reconstruction before hydrodynamic testing. The lower end of the VitaFlow frame was used as the THV reference. Because the surgical sewing ring is anatomically irregular and not perfectly planar, implantation depth was measured as the distance between the lower end of the THV frame and the bottom plane of the surgical valve sewing ring at multiple circumferential locations. The average of 5 measurements was considered the final implantation depth. Positive values indicate ventricular/subannular implantation below the sewing-ring plane, whereas negative values indicate supra-annular implantation above the sewing-ring plane toward the aortic side. Subsequently, in vitro hemodynamic testing was performed with multiple implantation depths of the THV device. Table 1 lists the true internal diameters of 21 mm SHVs based on the manufacturer's model.

**Table 1:** Structural Features of the SHVs Included in the Study

Manufacturer	Model	True ID	Type	Leaflet Material
Medtronic, Minneapolis, MN	Hancock II	17.0 mm	Supra-annular	Porcine
Medtronic, Minneapolis, MN	Mosaic	17.0 mm	Supra-annular	Porcine
Edwards Lifesciences, Irvine, CA	Perimount	19.0 mm	Intra-annular	Bovine
Edwards Lifesciences, Irvine, CA	Magna ease	19.0 mm	Supra-annular	Bovine
St Jude Medical, St Paul, MN	Epic	16.5 mm	Intra-annular	Porcine

### In vitro hemodynamic measurements

The ViV setups were tested at a temperature of 37 °C in a custom-built pulse duplicator system. Heart rate, blood pressure, and cardiac output were used as control parameters for the waveform generator controlling a servo pump. Pressures were measured in the left atrium, left ventricle, left ventricular outflow tract, and ascending aorta with strain gauge pressure transducers (Deltran; Utah Medical Products, Inc). An electromagnetic flowmeter (CME 500 Series; Carolina Medical Electronics Inc) was used to measure the aortic valve flow rate. In addition, normal saline solution was used as recirculating fluid for the hemodynamic measurements. Pulse duplicator input parameters were used to match the ISO 5840-1:2021 standard and Food and Drug Administration specifications for testing heart valves: heart rate of 70 beats/min, 35% systolic duration of cycle period, mean atrial and aortic pressures of 10 and 100 mm Hg, and cardiac output of 5 L/min. These hemodynamic parameters were maintained constant throughout the study. Mean gradient, effective orifice area (EOA), regurgitation volume, and valvular energy loss were determined by the pulse duplicator software. EOA was calculated using the Gorlin equation. In addition, short-axis images of the opening and closing of the devices were recorded by a high-speed camera (FASTCAM MINI WX50; Photron USA, Inc) during the entire cardiac cycle at a rate of 1,000 frames per second. The pinwheeling index was quantified from en face images by measuring the leaflet free-edge lengths and comparing them with the corresponding unconstrained ideal configurations, as previously described.

### Accelerated wear testing

Because THV underexpansion and leaflet deformation may adversely affect long-term durability in ViV procedures, accelerated wear testing (AWT) was performed following hydrodynamic assessment. A 21-mm VitaFlow valve and a 23-mm CoreValve Evolut PRO valve were implanted within nondegenerated, a 21-mm Hancock II bioprosthesis and a 21-mm Perimount bioprosthesis, respectively. Before ViV implantation, each THV underwent a complete loading and deployment process to fully simulate clinical transcatheter implantation. For each configuration, the THV was implanted at the optimal implantation depth identified by hydrodynamic testing and reference.

AWT was performed in accordance with ISO 5840-3: 2021 recommendations. Each valve underwent 200 million accelerated cycles, corresponding to approximately 5 years of simulated in vivo durability. Valve integrity was periodically assessed throughout testing, including evaluation for leaflet tear, perforation, stent-frame fracture, leaflet delamination, and abnormal leaflet motion.

### Statistical analysis

Statistical analyses were performed using IBM SPSS Statistics (Version 29.0, IBM Corp). Figures were generated using GraphPad Prism (Version 10.0, GraphPad Software). Continuous variables are presented as mean ± SD. No categorical variables were analyzed in this benchtop experimental study. Hydrodynamic measurements obtained from ten consecutive cardiac cycles were averaged before statistical analysis. Consequently, only one averaged observation from each implantation-depth configuration entered the statistical analyses.

Associations between implantation depth and mean pressure gradient, effective orifice area, regurgitation volume, total energy loss, and pinwheeling index were evaluated using polynomial regression models. Polynomial regression models were constructed using conventional (raw) polynomial terms to characterize nonlinear depth-response relationships. Quadratic and cubic polynomial models were evaluated for each outcome variable and compared according to adjusted R^2^, residual inspection, and analysis-of-variance comparison of nested models. The simplest model providing adequate fit was selected.

Analysis of variance was performed to evaluate the overall significance of the polynomial regression models. Because analysis of variance was used for regression model evaluation rather than multiple-group comparisons, post hoc testing and correction for multiple comparisons were not applicable. Two-sided *P* values < 0.05 were considered statistically significant. Where appropriate, fitted regression curves are presented together with mean ± SD error bars.

### Ethics approval or exemption

This in vitro benchtop experimental study used commercially available devices and did not involve human participants, animals, identifiable patient data, or human tissue. The Ethics Committee of Tianjin Chest Hospital determined that formal ethics approval was not required.

## Results

### ViV implantation characteristics

A total of 55 in vitro ViV implantations were performed using the 21-mm VitaFlow THV within five 21-mm SHVs. Implantation depth ranged from −1.0 to 16.5 mm below the SHV frame and was confirmed by CT reconstruction before hydrodynamic testing (Figure 2). Across all SHVs, progressive subannular implantation resulted in increasing THV frame constraint, leaflet distortion, and pinwheeling, particularly at deeper implantation depths.

**Figure 2:**
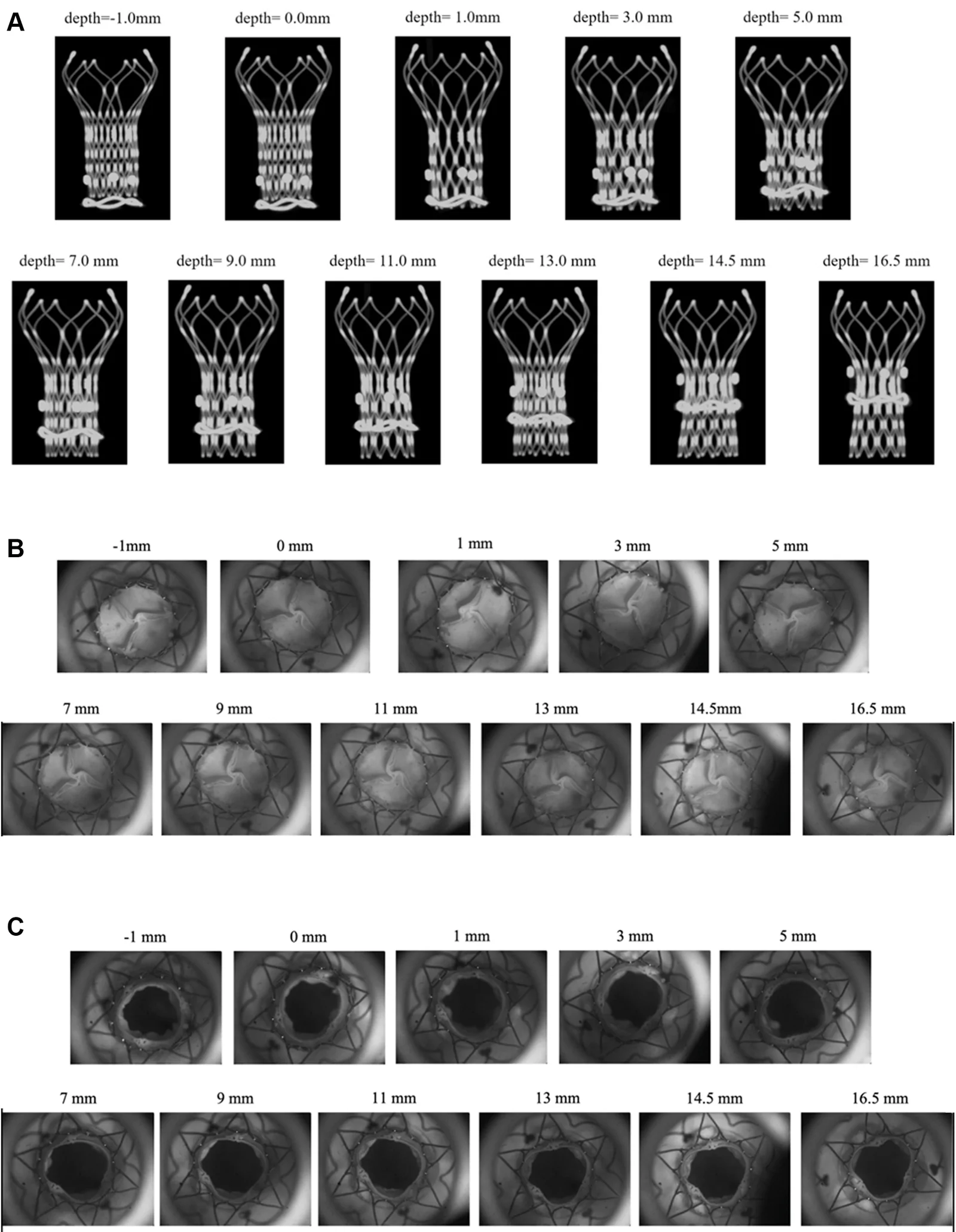
Representative Constrained Valve-in-Valve Configuration A 21-mm self-expanding transcatheter heart valve (THV) was implanted within a 21-mm surgical bioprosthesis with a true internal diameter of 17 mm. (A) Computed tomography reconstruction shows implantation depth and the spatial relationship between the THV frame and the surgical valve. Representative images during valve closure (B) and opening (C) demonstrate frame constraint and leaflet deformation under constrained valve-in-valve conditions.

### Hemodynamic performance according to SHV type

In Hancock II and Mosaic bioprostheses, moderate-to-deep implantation was associated with progressive improvement in forward-flow hemodynamics (Figures 3A and 3B), with up to 50% lower gradients and 30% higher EOA. Mean gradients decreased significantly with moderate-to-deep implantation (Hancock II: 51.9 ± 0.54 mm Hg to 27.5 ± 0.46 mm Hg, *P* = 0.003; Mosaic: 38.7 ± 0.35 mm Hg to 19.2 ± 0.26 mm Hg, *P* = 0.013), whereas EOA increased correspondingly (Hancock II: 1.0 ± 0.00 cm^2^ to 1.3 ± 0.01 cm^2^; Mosaic: 1.0 ± 0.01 cm^2^ to 1.2 ± 0.01 cm^2^; both *P* < 0.001). Regurgitation volume and valvular energy loss also decreased with progressive subannular implantation, with the lowest values observed at implantation depths of approximately 13.0 to 14.5 mm. Pinwheeling index progressively increased at deeper implantation depths in both Hancock II and Mosaic valves, indicating increasing leaflet torsion and THV frame constraint. Visual assessment similarly demonstrated increasing leaflet distortion and pinwheeling at deeper implantation depths, whereas higher implantation improved leaflet coaptation.

**Figure 3:**
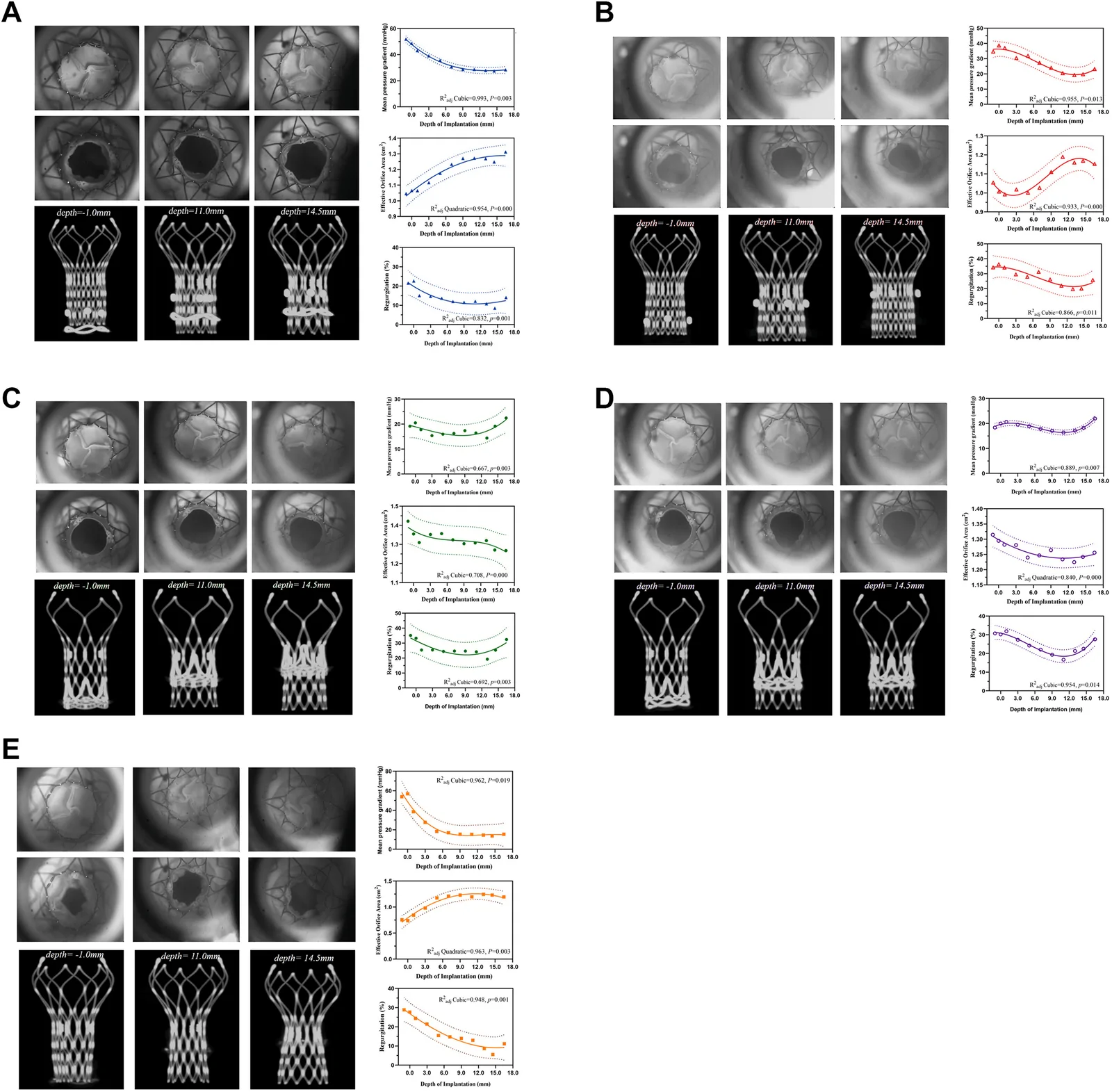
Depth-Dependent Valve-in-Valve (ViV) Configuration and Hemodynamics Representative valve-in-valve (ViV) configurations at different implantation depths are shown for 5 surgical heart valves (SHVs) platforms. For each SHVs, fluoroscopic images during valve closure and opening, reconstructed transcatheter heart valve (THV) frame geometry, and depth-response curves for mean gradient, effective orifice area, and regurgitation are presented. Implantation depth influenced both THV frame constraint and hydrodynamic performance, although the magnitude and pattern of response varied among SHVs types. These findings demonstrate that ViV hemodynamics are highly dependent on the interaction between implantation depth and surgical valve geometry. A: Hancock II, B: Mosaic, C: Perimount 2800; D: Magna Ease; E: Epic.

In contrast, Perimount bioprostheses demonstrated a distinct hemodynamic profile (Figure 3C). Mean gradient varied significantly across implantation depths (*P* = 0.003), reaching its lowest value at approximately 13.0 mm (14.4 ± 0.28 mm Hg) and increasing again at the deepest implantation depth of 16.5 mm (22.4 ± 0.66 mm Hg). Higher implantation was associated with larger EOA (1.3 ± 0.01 cm^2^ to 1.5 ± 0.01 cm^2^; *P* < 0.001), whereas deeper implantation remained associated with lower regurgitation volume and reduced valvular energy loss, with nadir values near 13.0 mm implantation depth. Compared with the porcine bioprostheses, the Perimount and Magna ease valves demonstrated less severe THV underexpansion, leaflet deformation, and pinwheeling, consistent with their larger true internal diameter and lower degree of THV oversizing (Figure 3D).

Among all SHVs tested, Epic bioprostheses demonstrated the widest range of transvalvular gradients across implantation depths (Figure 3E). Mean gradient decreased substantially with deeper implantation, ranging from 56.9 ± 0.50 mm Hg to 13.8 ± 0.24 mm Hg (*P* = 0.019), whereas EOA increased from 0.7 ± 0.01 cm^2^ to 1.2 ± 0.01 cm^2^ (*P* = 0.003). Regurgitation volume and valvular energy loss similarly decreased with deeper implantation, with optimal values observed between 13.0 and 14.5 mm. Pinwheeling index progressively increased with deeper implantation depth, paralleling the increase in THV frame constraint and leaflet distortion, whereas higher implantation improved leaflet coaptation.

Total valvular energy loss decreased with deeper implantation depth across all 5 SHV platforms (Central Illustration, Table 2). The lowest energy loss values consistently occurred at implantation depths between approximately 13.0 and 14.5 mm, corresponding to the level at which the outer skirt was fully located beneath the surgical valve frame.

**Central Illustration:**
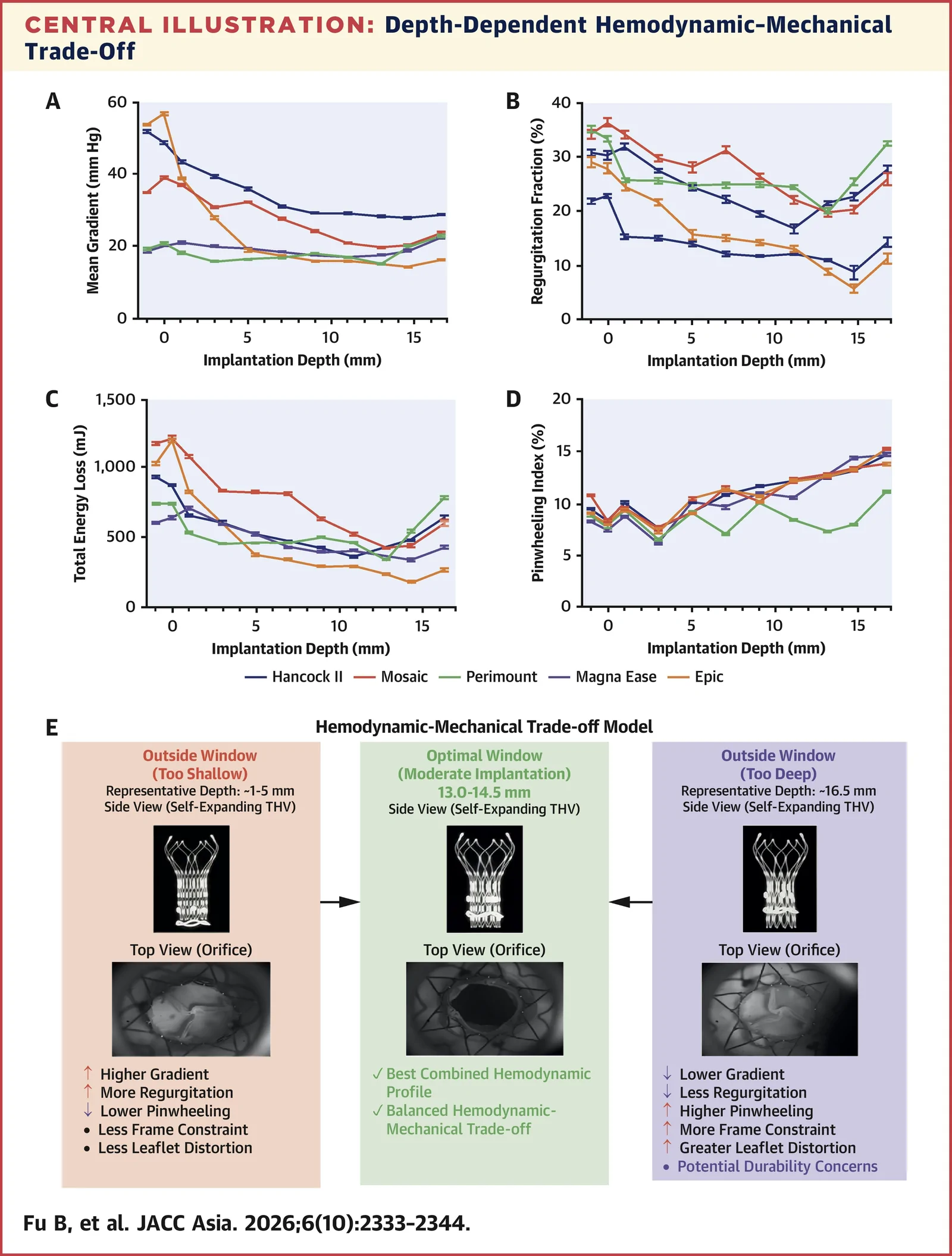
Depth-Dependent Hemodynamic–Mechanical Trade-Off Mean transvalvular gradient (A), regurgitation fraction (B), total energy loss (C), and pinwheeling index (D) are shown across implantation depths in 5 small surgical bioprosthetic valves implanted with a 21-mm self-expanding transcatheter heart valve. Implantation depth was analyzed from −1 to 16.5 mm. Across valve types, increasing implantation depth was generally associated with improved hydrodynamic performance, reflected by reductions in mean gradient, regurgitation fraction, and total energy loss over a moderate depth range. However, the pinwheeling index, a surrogate of leaflet deformation, showed an overall depth-dependent increase at deeper implantation levels, indicating greater leaflet distortion and mechanical constraint despite improved hydrodynamic parameters. The conceptual model (E) summarizes the trade-off between improved hydrodynamic performance and increased leaflet deformation, frame constraint, and pinwheeling at moderate-to-deep implantation depths.

**Table 2:** Compiled Results

Deployment	Mean Gradients (mm Hg)	Effective Orifice Area (cm^2^)	Regurgitant Fraction (%)	Total Energy Loss (mJ)	Pinwheeling Index (%)
Hancock II					
−1 mm	51.9 ± 0.54	1.1 ± 0.00	21.6 ± 0.72	932.4 ± 13.29	8.4 ± 0.12
0 mm	48.6 ± 0.51	1.1 ± 0.01	22.6 ± 0.46	877.0 ± 9.47	7.6 ± 0.15
1 mm	43.1 ± 0.65	1.1 ± 0.01	15.1 ± 0.55	657.3 ± 10.42	8.9 ± 0.13
3 mm	39.1 ± 0.50	1.1 ± 0.01	14.7 ± 0.54	594.5 ± 9.64	6.3 ± 0.18
5 mm	35.7 ± 0.59	1.2 ± 0.01	13.7 ± 0.74	534.6 ± 8.24	10.2 ± 0.15
7 mm	30.7 ± 0.36	1.2 ± 0.01	11.9 ± 0.48	430.8 ± 7.21	9.7 ± 0.26
9 mm	28.8 ± 0.31	1.3 ± 0.00	11.5 ± 0.32	402.7 ± 4.92	11.1 ± 0.13
11 mm	28.7 ± 0.54	1.3 ± 0.01	12.1 ± 0.45	410.8 ± 7.60	10.6 ± 0.21
13 mm	27.9 ± 0.47	1.3 ± 0.01	10.8 ± 0.30	380.4 ± 4.67	12.8 ± 0.20
14.5 mm	27.5 ± 0.46	1.2 ± 0.01	8.5 ± 1.86	343.8 ± 16.03	14.3 ± 0.17
16.5 mm	28.4 ± 0.26	1.3 ± 0.01	14.1 ± 1.01	431.9 ± 12.4	14.6 ± 0.19
Mosaic					
−1 mm	34.7 ± 0.34	1.0 ± 0.00	34.2 ± 1.24	1,025.8 ± 10.64	9.2 ± 0.11
0 mm	38.7 ± 0.35	1.0 ± 0.01	36.4 ± 1.11	1,185.8 ± 20.49	8.1 ± 0.14
1 mm	37.0 ± 0.58	1.0 ± 0.00	34.1 ± 1.06	1,078.3 ± 14.30	9.7 ± 0.13
3 mm	30.5 ± 0.38	1.0 ± 0.00	29.6 ± 0.82	833.7 ± 10.06	7.4 ± 0.14
5 mm	31.9 ± 0.33	1.0 ± 0.00	27.9 ± 1.40	825.1 ± 18.28	10.5 ± 0.20
7 mm	27.2 ± 0.23	1.0 ± 0.00	31.2 ± 0.87	811.6 ± 16.18	11.3 ± 0.15
9 mm	23.9 ± 0.30	1.1 ± 0.00	26.2 ± 0.79	633.6 ± 10.93	10.8 ± 0.18
11 mm	20.4 ± 0.30	1.2 ± 0.00	21.9 ± 1.04	523.5 ± 13.35	12.1 ± 0.17
13 mm	19.2 ± 0.26	1.2 ± 0.00	19.7 ± 1.04	426.3 ± 11.51	12.7 ± 0.21
14.5 mm	19.8 ± 0.28	1.2 ± 0.00	20.1 ± 1.16	443.9 ± 17.36	13.4 ± 0.11
16.5 mm	23.2 ± 0.37	1.1 ± 0.02	25.8 ± 1.77	603.5 ± 25.77	13.8 ± 0.19
Perimount					
−1 mm	19.2 ± 0.28	1.4 ± 0.01	35.2 ± 0.82	737.9 ± 10.37	8.9 ± 0.11
0 mm	20.5 ± 0.39	1.3 ± 0.01	33.3 ± 0.78	741.9 ± 12.43	7.8 ± 0.11
1 mm	17.8 ± 0.46	1.3 ± 0.01	25.4 ± 0.46	537.5 ± 7.35	9.6 ± 0.11
3 mm	15.4 ± 0.33	1.4 ± 0.01	25.5 ± 0.75	460.1 ± 7.31	6.7 ± 0.12
5 mm	15.9 ± 0.34	1.4 ± 0.01	24.5 ± 0.61	465.9 ± 4.90	9.3 ± 0.12
7 mm	16.3 ± 0.24	1.3 ± 0.01	24.7 ± 0.57	466.7 ± 6.57	7.2 ± 0.12
9 mm	17.4 ± 0.46	1.3 ± 0.01	24.7 ± 0.69	501.6 ± 7.95	10.1 ± 0.12
11 mm	16.4 ± 0.27	1.3 ± 0.01	24.2 ± 0.46	466.7 ± 5.84	8.5 ± 0.11
13 mm	14.4 ± 0.28	1.3 ± 0.01	19.2 ± 0.46	345.4 ± 6.78	7.5 ± 0.13
14.5 mm	19.2 ± 0.40	1.2 ± 0.01	25.3 ± 0.87	546.0 ± 12.58	8.1 ± 0.11
16.5 mm	22.4 ± 0.66	1.2 ± 0.01	32.4 ± 0.53	784.7 ± 13.23	11.2 ± 0.13
Manga ease					
−1 mm	18.4 ± 0.46	1.3 ± 0.01	30.7 ± 0.79	607.5 ± 9.34	9.6 ± 0.11
0 mm	19.9 ± 0.27	1.3 ± 0.01	30.2 ± 1.13	640.4 ± 13.36	8.4 ± 0.12
1 mm	20.6 ± 0.49	1.3 ± 0.01	31.9 ± 1.00	707.7 ± 15.19	10.1 ± 0.12
3 mm	19.5 ± 0.42	1.3 ± 0.02	27.3 ± 0.48	610.2 ± 15.91	7.8 ± 0.12
5 mm	18.9 ± 0.33	1.2 ± 0.01	24.2 ± 0.78	521.8 ± 10.39	9.3 ± 0.12
7 mm	17.8 ± 0.25	1.2 ± 0.01	22.0 ± 1.04	474.9 ± 9.69	10.9 ± 0.11
9 mm	17.0 ± 0.35	1.3 ± 0.01	19.3 ± 0.76	429.6 ± 9.34	11.7 ± 0.11
11 mm	16.4 ± 0.17	1.2 ± 0.01	16.6 ± 1.19	364.6 ± 15.03	12.2 ± 0.10
13 mm	17.1 ± 0.21	1.2 ± 0.01	21.3 ± 0.44	423.9 ± 8.07	12.5 ± 0.10
14.5 mm	18.2 ± 0.30	1.2 ± 0.01	22.5 ± 0.99	484.0 ± 13.59	13.1 ± 0.11
16.5 mm	21.9 ± 0.27	1.2 ± 0.01	27.6 ± 0.86	645.0 ± 19.70	14.7 ± 0.12
Epic					
−1 mm	53.9 ± 0.39	0.7 ± 0.00	28.9 ± 1.31	1,171.6 ± 16.58	10.8 ± 0.10
0 mm	56.9 ± 0.50	0.7 ± 0.01	27.7 ± 1.22	1,202.7 ± 19.23	8.6 ± 0.10
1 mm	38.6 ± 0.47	0.8 ± 0.00	24.4 ± 1.16	822.2 ± 14.96	9.4 ± 0.12
3 mm	27.6 ± 0.57	0.9 ± 0.01	21.4 ± 0.74	598.3 ± 8.80	7.9 ± 0.11
5 mm	18.3 ± 0.44	1.2 ± 0.01	15.5 ± 1.31	376.2 ± 15.56	9.1 ± 0.12
7 mm	16.9 ± 0.24	1.2 ± 0.01	14.8 ± 0.68	342.9 ± 8.35	11.6 ± 0.14
9 mm	15.4 ± 0.26	1.2 ± 0.01	13.9 ± 0.74	297.5 ± 8.69	10.2 ± 0.13
11 mm	15.3 ± 0.32	1.2 ± 0.01	12.9 ± 0.86	292.4 ± 9.69	12.4 ± 0.11
13 mm	14.6 ± 0.22	1.2 ± 0.01	8.7 ± 0.78	235.2 ± 9.58	12.9 ± 0.12
14.5 mm	13.7 ± 0.24	1.2 ± 0.01	5.6 ± 1.10	183.9 ± 10.47	13.2 ± 0.11
16.5 mm	15.5 ± 0.29	1.2 ± 0.02	11.2 ± 1.33	267.0 ± 16.94	15.1 ± 0.12

### AWT assessment following ViV implantation

Because moderate-to-deep implantation was associated with increased THV frame constraint, leaflet deformation, and pinwheeling, AWT was performed to evaluate the potential durability implications of ViV underexpansion. Despite constrained THV expansion within the surgical bioprostheses, the selected ViV configurations successfully completed 200 million accelerated wear cycles without evidence of structural valve deterioration, leaflet tear, stent-frame fracture, leaflet delamination, or abnormal leaflet motion (Figure 4).

**Figure 4:**
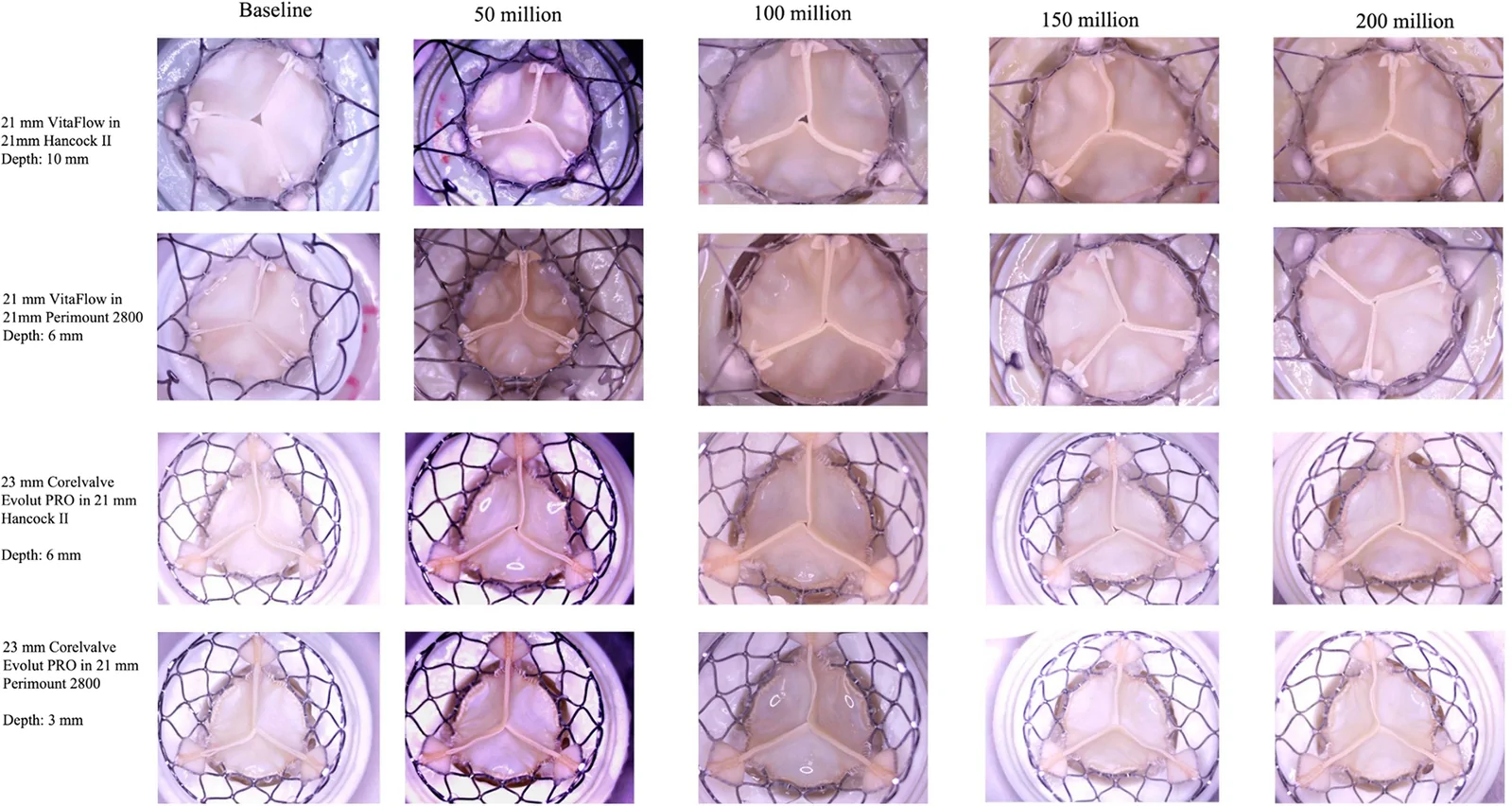
Accelerated Wear Testing Accelerated wear testing was performed under ISO 5840 conditions in selected constrained valve-in-valve configurations. Both tested configurations completed 200 million accelerated cycles without gross structural failure, including leaflet tear, stent-frame fracture, leaflet delamination, or abnormal leaflet motion. These findings support preserved gross structural integrity under bench conditions but do not establish long-term clinical durability.

Although these results suggest preserved mechanical performance under the tested conditions, they should be interpreted as preclinical evidence of structural integrity rather than proof of long-term clinical durability.

## Discussion

The principal findings of this study are summarized in the Central Illustration. First, implantation depth substantially influenced ViV hemodynamics in small surgical bioprostheses. Second, the depth-response relationship was not linear and varied according to surgical valve characteristics. Third, selected porcine surgical bioprostheses demonstrated a moderate-to-deep implantation window associated with the most favorable combined hemodynamic profile, whereas bovine pericardial bioprostheses showed more heterogeneous or U-shaped responses. Fourth, deeper implantation increased THV frame constraint, leaflet deformation, and pinwheeling, indicating a trade-off between acute hydrodynamic improvement and mechanical deformation burden. Finally, selected constrained ViV configurations completed 200 million accelerated wear cycles without gross structural failure under ISO 5840 conditions; however, these findings should be interpreted as evidence of preserved structural integrity under bench conditions rather than proof of long-term clinical durability.

### Depth-window concept in small ViV-TAVR

Prior in vitro and clinical studies have shown that implantation depth, THV type, and valve size substantially influence ViV hemodynamics.^8^, ^9^, ^10^, ^11^ The present findings support a mechanistic trade-off model. In selected small SHVs, deeper implantation may improve hemodynamics by reducing paravalvular leakage, skirt-related energy loss, and leaflet-flow interference.

The key finding is not that deeper implantation is universally beneficial, but rather that each THV-SHV combination may possess a finite implantation-depth window within which the overall balance between forward-flow hemodynamics and mechanical deformation is optimized. In selected porcine surgical bioprostheses, moderate-to-deep implantation was associated with lower gradients, larger effective orifice area, and reduced energy loss. Beyond this range, however, progressive frame constraint was accompanied by greater leaflet deformation and pinwheeling. These observations indicate that implantation depth should be determined by the best combined profile of gradient, EOA, regurgitation, energy loss, and leaflet deformation rather than by any single hemodynamic parameter.

Importantly, the implantation-depth ranges identified in this study should be regarded as valve-combination-specific observations derived from controlled bench testing rather than universal procedural targets. The findings therefore support a depth-window concept rather than a “deeper-is-better” strategy.

### Mechanistic basis of valve-combination specificity

The observed differences among surgical bioprostheses provide insight into the mechanisms governing ViV performance. Porcine surgical valves generally demonstrated a clearer depth-response pattern, whereas bovine pericardial valves exhibited more heterogeneous behavior. These differences likely reflect variation in true internal diameter, valve geometry, and the degree of THV oversizing.

Surgical bioprostheses with smaller true internal diameters imposed greater constraint on THV expansion, resulting in more pronounced frame deformation, leaflet distortion, and pinwheeling. Conversely, larger internal diameters reduced the degree of oversizing and mechanical constraint. These findings suggest that ViV performance is determined by the interaction among implantation depth, THV structural characteristics, and surgical valve geometry rather than by implantation depth alone.

Structural differences among contemporary self-expanding THV platforms may further influence these relationships. However, the present study was not designed as a head-to-head comparison of different THVs. Therefore, the mechanistic observations reported here should not be generalized across valve platforms without further validation.

### AWT and durability interpretation

Leaflet pinwheeling and THV underexpansion are increasingly recognized as potential markers of abnormal leaflet stress and durability impairment.^15^ Prior computational work has shown that TAV underexpansion can distort leaflet configuration, increase leaflet stress, and accelerate fatigue damage, particularly when underexpansion reaches ≥2 mm or ≥9.1% of valve diameter.^16^ This provides a mechanistic rationale for evaluating durability in the present study, because deeper implantation improved hemodynamics but also increased pinwheeling and leaflet deformation, particularly in valves with smaller true internal diameters.

Despite constrained THV expansion, the selected ViV configurations completed 200 million ISO 5840 accelerated wear cycles without evidence of leaflet tear, frame fracture, leaflet delamination, or abnormal leaflet motion. These findings indicate preserved gross structural integrity under standardized bench conditions. However, they should not be interpreted as evidence of long-term clinical durability.

AWT cannot reproduce important biological processes encountered in vivo, including thrombosis, hypoattenuated leaflet thickening, calcification, inflammation, tissue degeneration, or patient-specific loading conditions. Consequently, the absence of structural failure during bench testing does not exclude the possibility of leaflet dysfunction or structural valve deterioration during long-term clinical follow-up. Dedicated imaging studies and larger durability investigations remain necessary.

### Clinical implications

The present findings have several clinical implications. First, implantation strategy in small ViV procedures should consider not only supra-annular positioning principles but also the interaction between THV design and surgical valve geometry.^17^ Second, acute hemodynamic optimization should be balanced against progressive mechanical deformation, particularly in highly constrained valve configurations.^18^ Third, implantation-depth planning should be individualized according to the specific THV-SHV combination rather than guided by a uniform depth target.

Importantly, the implantation-depth windows identified in this study should be viewed as mechanistic and hypothesis-generating rather than prescriptive procedural recommendations. In clinical practice, implantation depth represents only 1 component of procedural planning and must be integrated with considerations of coronary obstruction risk, anchoring stability, commissural alignment, conduction disturbances, and patient-specific anatomy.^19^^,^^20^ Representative clinical cases illustrating the relevance of implantation depth in small ViV procedures are shown in Figure 5.The representative clinical cases illustrate that implantation depths falling within the bench-derived depth window were achievable during routine ViV-TAVR procedures and were associated with acceptable postprocedural gradients. Although these observations should not be considered validation, they support the procedural feasibility of the proposed depth-window concept.

**Figure 5:**
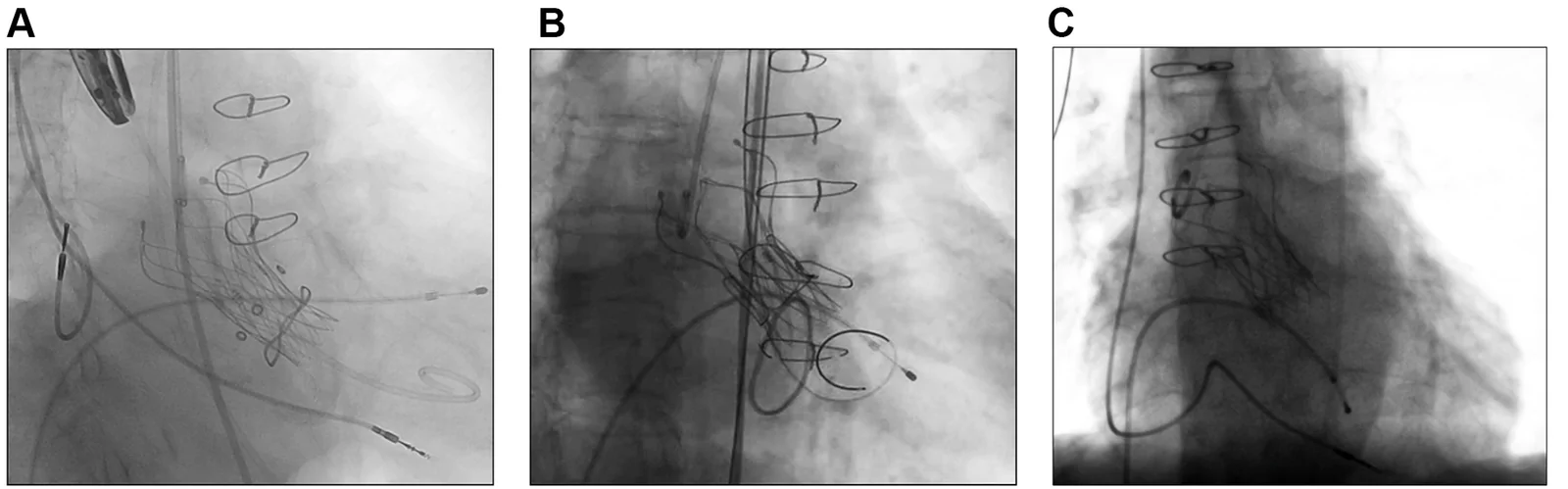
Representative Clinical Valve-in-Valve Cases Representative transfemoral valve-in-valve procedures in small surgical bioprostheses. (A) Porcine bioprosthesis (true internal diameter, 17 mm), implantation depth 11 mm; mean gradient decreased from 36.2 to 17.6 mm Hg. (B) Porcine surgical bioprosthesis (true internal diameter, 19 mm), implantation depth 11 mm; mean gradient decreased from 41.4 to 11.7 mm Hg. (C) Bovine pericardial bioprosthesis (true internal diameter, 19 mm), implantation depth 6 mm; mean gradient decreased from 13.0 to 10.0 mm Hg.

### Study limitations

First, this was an in vitro bench study using selected THV-SHV combinations under controlled hydrodynamic conditions; therefore, the results may not fully reproduce patient-specific anatomy, native root compliance, coronary sinus dynamics, blood viscosity, thrombogenicity, calcification, hypoattenuated leaflet thickening, inflammation, or biologic leaflet degeneration. Second, AWT was limited to selected constrained ViV configurations and should be interpreted as preclinical evidence of preserved gross structural integrity under ISO 5840 conditions, not proof of long-term clinical durability. Third, although Figure 1 illustrates relevant structural differences among contemporary self-expanding THV platforms, the present study was not designed as a head-to-head hemodynamic comparison across different THVs. Fourth, the proposed implantation-depth windows are specific to the tested valve models, sizes, and bench setup and should not be generalized as universal implantation targets without clinical validation. Future studies should include larger head-to-head THV comparisons across matched SHV types and implantation-depth windows, computational stress analysis, micro-CT assessment of frame geometry, and longitudinal CT and echocardiographic follow-up after clinical ViV-TAVR.

## Conclusions

Implantation depth substantially influenced ViV hemodynamics in small surgical bioprostheses, but the depth-response relationship was not linear. Selected porcine surgical bioprostheses demonstrated a moderate-to-deep implantation window associated with the most favorable combined hemodynamic profile, whereas bovine pericardial bioprostheses showed more heterogeneous responses. Deeper implantation depth was also associated with increased THV frame constraint, leaflet deformation, and pinwheeling, supporting a mechanistic trade-off between acute hydrodynamic improvement and mechanical deformation burden. The implantation-depth ranges identified in this study should therefore be interpreted as valve-combination-specific bench observations rather than universal implantation targets. Selected constrained ViV configurations maintained gross structural integrity after 200 million ISO 5840 accelerated wear cycles; however, these findings do not establish clinical durability and require validation in larger bench studies and longitudinal clinical imaging follow-up.

## Funding Support and Author Disclosures

This work was supported by the Tianjin Health Research Project (Grant No. TJWJ2026XK026); the Tianjin Key Medical Discipline Construction Project (Grant No. TJYXZDXK-3-030C); and the National Engineering Research Center of Science and Technology Information STI Digital Health Laboratory (Beijing Zhilian Research Institute for Innovation and Digital Health; Grant No. 2024STI044). Tianjin Key Laboratory of Cardiovascular Emergency and Critical Care, Tianjin Municipal Science and Technology Bureau. The authors have reported that they have no relationships relevant to the contents of this paper to disclose.
